# Mesenteric excision surgery or conservative limited resection in Crohn’s disease: study protocol for an international, multicenter, randomized controlled trial

**DOI:** 10.1186/s13063-020-4105-x

**Published:** 2020-02-21

**Authors:** Yi Li, Helen Mohan, Nan Lan, Xiaojian Wu, Wei Zhou, Jianfeng Gong, Bo Shen, Luca Stocchi, J. Calvin Coffey, Weiming Zhu

**Affiliations:** 1 Center for Inflammatory Bowel Diseases, Department of General Surgery, Jinling Hospital, Medical School of Nanjing University, Nanjing, Jiangsu China; 2 Department of Surgery, Surgical Professorial Unit, University Hospital Limerick, Limerick, Ireland; 3 Department of Colorectal Surgery, Digestive Disease and Surgery Institute Cleveland Clinic, 9500 Euclid Avenue, A30, Cleveland, OH 44195-0001 USA; 4 Department of Colorectal Surgery, the Sixth Affiliated Hospital of Sun Yat-sen University, Guangzhou, Guangdong China; 5 Department of General Surgery, Sir Run Run Shaw Hospital, School of Medicine, Zhejiang University, Zhejiang, Hangzhou China; 6 Section of Inflammatory Bowel Diseases and Center for Interventional IBD, Columbia University Irving Medical Center-NewYork Presbyterian, New York, NY USA

**Keywords:** Crohn’s disease, Ileocolic resection, Mesentery, Postoperative recurrence

## Abstract

**Background:**

The structures of the mesentery including adipose tissue, nerves, and lymphatics play an important role in the pathogenesis and disease progression of Crohn’s disease (CD). Conventional surgical resection for CD usually does not involve resecting the mesentery en bloc with the specimen. This contrasts with complete mesocolic excision (CME) in colorectal cancer, which involves radical resection of the mesentery. Preliminary evidence from smaller studies suggests that applying the principle of mesocolic excision to CD surgery may reduce the risk of postoperative recurrence. This randomized controlled trial is designed to test whether applying the principles of mesocolic excision to CD results in reduced postoperative recurrence. It also aims to evaluate intra- and postoperative morbidity between the two approaches.

**Methods:**

This international, multicenter, randomized controlled trial will randomize patients (*n* = 116) scheduled to undergo primary ileocolic resection to either receive extensive mesenteric excision (EME) or conventional ileocolic resection with limited mesenteric excision (LME). Five sites will recruit patients in three countries. In the EME group, the mesentery is resected following CME, while avoiding the root region, i.e., 1 cm from the root of the ileocolic artery and vein. In the LME group, the mesentery is retained, i.e., “close shave” or < 3 cm from the border of bowel. The primary end point will be surgical recurrence after surgery. The secondary end points will be the postoperative endoscopic and clinical recurrence, and intra- and postoperative morbidity. Demographics, risk factors, laboratory investigations, endoscopy, postoperative prophylaxis and imaging examination will be assessed. Analysis of the primary outcome will be on an intention-to-treat basis.

**Discussion:**

If mesocolic excision in CD reduces postoperative disease recurrence and does not increase morbidity, this trial has the potential to change practice and reduce recurrence of CD after surgical resection.

**Trial registration:**

Clinical Trials.gov, ID: NCT03769922. Registered on February 27, 2019.

## Background

Disease recurrence at the site of the anastomosis after ileocolic resection for Crohn’s disease (CD) is of pivotal clinical importance. More than 90% of CD patients develop endoscopic recurrence, 20% of whom are symptomatic. Despite developments in biological therapy for CD, approximately 30–40% of patients require reoperation within the first 5 to 10 years. In addition to environmental factors, such as smoking and diet, the clinical pattern of disease, e.g., stricturing versus fistulating behavior, and pathological features of the resection margin, may also influence the risk of postoperative recurrence [1, 2].

Abnormal changes in the mesentery in patients with CD have been noted for several decades. Emerging data suggest that the mesenteric tissue plays an important role in the pathogenesis of CD [3–5]. A high value of visceral fat content [6], an increased mesenteric lymphatic-vessel density in the resection margin [7], and the presence of granulomata in the mesenteric lymph nodes have been found to be associated with postoperative recurrence [8].

Conventional surgical resection for CD does not usually involve resection of the associated mesentery, with traditional teaching advocating staying “close to the bowel wall.” To date, the inclusion of the mesentery in intestinal resection for CD has not become standard practice. This is largely due to technical concerns regarding risk of bleeding when handling tissues with significant inflammation, e.g., disease-related perforation, fistula formation, adhesions, and thickened mesentery [9, 10], which could lead to hematomas, ongoing hemorrhage, and injury to the mesenteric root [11, 12]. Therefore, despite recent interest in the role of the mesentery in CD, the implication of resection of the mesentery along with the diseased bowel has not been evaluated.

Our preliminary data indicate that: (1) extensive mesenteric excision is safe and feasible in surgical resection for CD; (2) inclusion of mesentery resection does not cause specific morbidity; and (3) extensive mesenteric excision results in reduced incidence of endoscopic recurrence after surgery (unpublished data). In addition, Coffey et al. reported that the inclusion of the mesentery in the course of ileocolic resection for CD is associated with reduced recurrence requiring reoperation [9].

To date, there has been no prospective trial comparing wide mesocolic resection with conventional surgery in CD. Thus, the objective of this multicenter, single-blind, randomized controlled trial is to determine whether there is a reduction in the rate of postoperative recurrence of CD following extensive mesenteric excision (EME), when compared to that of conventional limited mesenteric excision (LME) for ileocolic disease.

## Methods

### Study population

**Fig. 1:**
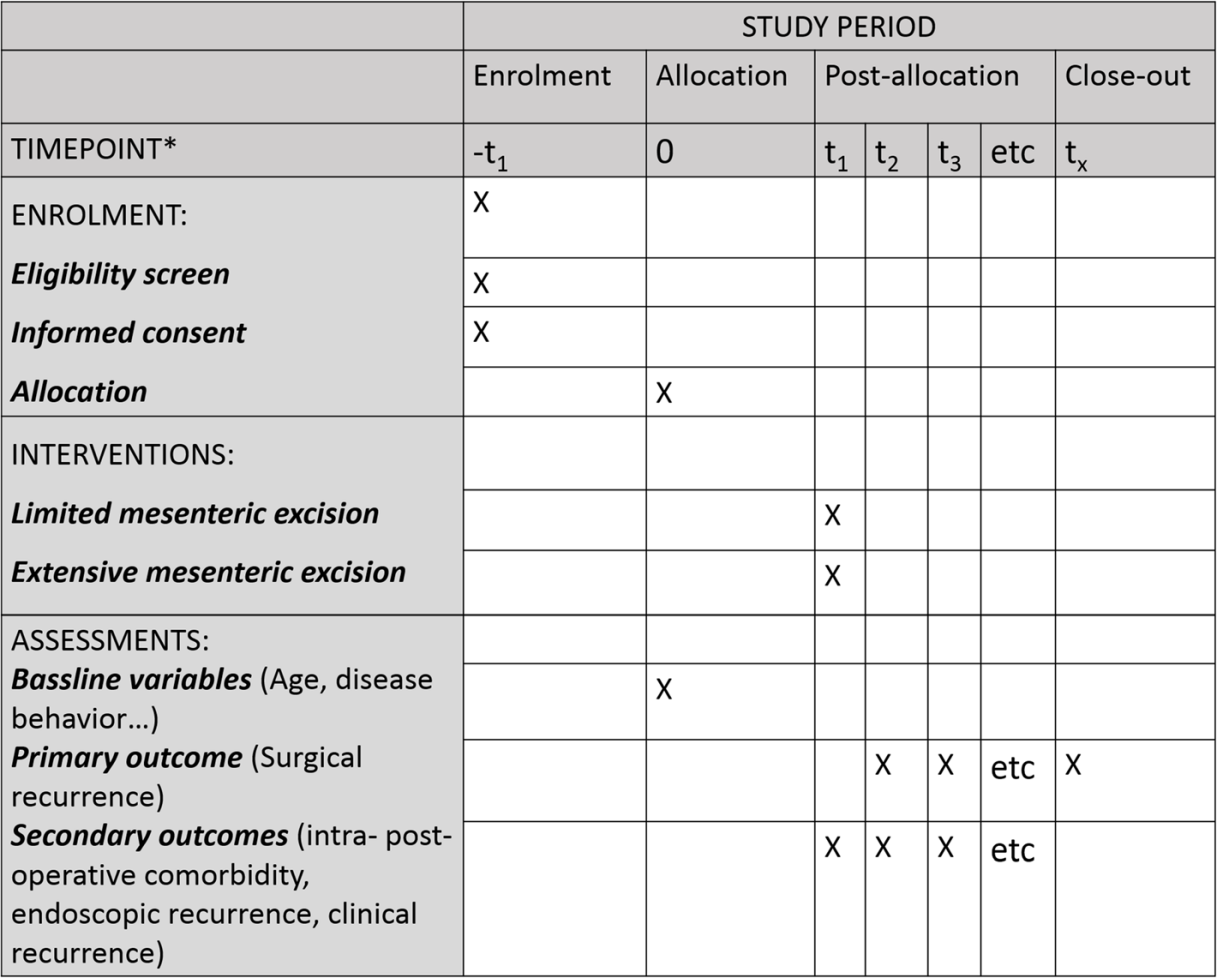
Standard Protocol Items: Recommendations for Interventional Trials (SPIRIT) Flow Diagram of schedule of enrollment, interventions, and assessments

Patients with CD limited to the distal ileum and/or right colon receiving their index ileocolic resection are eligible for inclusion. Eligibility criteria include: age between 18 and 65 years with a documented history of CD based on endoscopic, radiological, or histological criteria. The exclusion criteria are: pregnancy or planning to become pregnant in the following year; CD located at a gastrointestinal site other than the terminal ileal cecum, or right colon; and intestinal fistula requiring resection of another segment of bowel. Eligible patients with perianal disease will be included. All participants will provide written informed consent. The schedule of enrollment, interventions, and assessment is presented in Fig. 1 (see also Additional file 1: Standard Protocol Items: Recommendations for Interventional Trials (SPIRIT) Checklist LME EME).

### Randomization

Given the nature of surgical procedures, it is not possible to blind the surgical team to the assigned treatment arm. However, patients will be blinded to the type of mesenteric excision performed. Eligible patients enrolled by each participating center will be randomized based on a computer-generated list maintained centrally. The randomization codes are uploaded on Google docs, and the study co-ordinator at each center will receive a link to the codes.. The group allocation will be conducted by a staff surgeon, or research assistant who is blind to the study by a sealed, opaque envelope. Individuals other than the surgical team involved in the trial will be blind to the assigned treatment arm according to the blinding guidelines indicated by Probst et al. [13].

After entering the abdomen, the surgeon will determine whether a hemostatic division of the mesentery is technically possible. If this is the case, as we would expect in the majority of patients, the envelope containing the randomization can be opened to find out what group the patient has been randomized to, and the operation proceeds as per the group allocation to either a mesenteric-based resection or conventional resection. If mesenteric division is not deemed possible, the patient will be excluded from the trial. The reasons for those that have not been randomized would be clearly reported. The number of excluded patient will be separately recorded. Alternatively, the patient may undergo initial stoma diversion followed by delayed resection with preoperative re-randomization in 6 months. This approach will be left to the discretion of the operating surgeon, while the reason for stoma creation should be documented. Patients undergoing delayed resection are, therefore, eligible for inclusion in the study.

### Surgical procedure

Depending on patient and surgeon preference, surgery will be performed laparoscopically or open. Ileocolic mesenteric mobilization and hemostatic division of the mesentery will be performed as reported [9]. In a conventional limited resection, the mesentery is resected within 3 cm from the bowel, leaving most of the mesentery in situ. In the extensive mesenteric resection group, a mesocolic resection is performed while avoiding the root region by dividing the mesentery 1 cm from the root of ileocolic artery and vein. The proximal mesenteric incision will be made just proximal to the mesenteric transition zone [9], while the distal incision will be placed where both the mesentery and intestine are macroscopically normal immediately distal to the region of disease. After resection, an ileocolic anastomosis will be then performed. The type of anastomosis construction and the anastomotic configuration are based on the individual surgeon’s preference.

We expect that the majority of patients should be treated with ileocolic resection and primary anastomosis. However, in selected cases the surgeon may elect to reroute the fecal stream away from an anastomosis with proximal stoma diversion based on the surgeons’ clinical judgment and intra-operative findings. The reasons for stoma diversion should be clearly documented. If a covering loop-ileostomy is fashioned then it is recommended that this be reversed (if feasible) after 3 months and that the time of ileocolic resection and ileostomy be considered as the starting point of postoperative surveillance. However, the date of reversal will also be recorded as it is possible that recurrence will differ in those patients who undergo diversion compared to those who are not.

### Postoperative examination of the resection specimen

The mesentery will be inspected and morphological features of mesenteric disease will be recorded. Mesenteric and mucosal disease scores will be documented [9]. For histopathological assessment, **e**ach specimen will be examined at two levels, intestinal and mesenteric. Each will be examined at a number of points along the longitudinal axis of the intestine [9].

### Maintenance therapy

Prophylactic treatment is recommended after ileocolic intestinal resection in patients with at least one risk factor for recurrence (Table 1) [14]. For those developing endoscopic recurrence or clinical recurrence after surgery, management will be undertaken in keeping with local gastroenterology standard practice and guidelines.

**Table 1:** Predictors of early postoperative recurrence after ileocolonic resection

Risk factor	Level of evidence
Smoking	EL1
Prior intestinal surgery	EL1
Penetrating disease at index surgery	EL2
Perianal location	EL2
Granulomata in resection specimen	EL2
Myenteric plexitis	EL3

### End points

In total, patients will be followed for 5 years after surgery. The primary end point of the study is postoperative surgical recurrence. Surgical recurrence is defined as the requirement for repeat surgery for a CD-related indication caused by recurrent disease in the same anatomical areas. If reoperation is required it should be documented whether the reason is a specific CD-related complication, or a complication related to surgery in general (i.e., hemorrhage, intraperitoneal collection not amenable to conservative treatment, wound dehiscence, anastomotic leakage). CD-related indications for surgery include: (1) development of symptoms refractory to medical treatment; and (2) development of a complication caused by CD (i.e., fistula, perforation, and obstruction).

Secondary end points include assessment of endoscopic recurrence and clinical recurrence. Endoscopic recurrence will be assessed using the widely accepted Rutgeert’s score [15]. Only disease (Rutgeert’s score i2, or higher) proximal to the anastomosis or in the perianastomotic area is considered to be consistent with endoscopic recurrence. Disease in other sites will not be considered to be recurrence. Clinical recurrence is defined as the presence of endoscopic disease or radiological evidence plus the presence of symptoms attributable to CD that are severe enough to require medical or surgical treatment.

In addition, intra-operative complications and 30-day postoperative outcomes will be evaluated. These include estimated blood loss, duration of surgery, postoperative morbidity, reoperation rate, and the length of postoperative hospital stay. Postoperative complications will be recorded and classified based on the Clavien-Dindo classification [16].

### Follow-up

Postoperative reviews will be conducted at 6-monthly intervals at which point patients will have a full clinical, endoscopic, and radiological assessment. The CD Activity Index [17] will be also recorded at each visit. If individuals experience symptoms suggestive of recurrent CD prior to the scheduled colonoscopy, they will undergo a colonoscopy and other investigations as deemed appropriate by their own gastroenterologist or surgeon to ascertain whether they have recurrent disease. If endoscopic or clinical recurrence are demonstrated, the patient should undergo continued follow-up if, or until, they develop recurrence requiring surgery. If patients do develop surgical recurrence, their participation in the study is terminated as they are considered to have reached the primary and ultimate end point.

### Sample size

There are no registered or published randomized controlled trials (RCTs) comparing limited with extensive mesenteric resection in patients with CD. Our sample size calculation is based on the surgical recurrence in a retrospective study reported by Coffey et al. [9]. It is estimated that 30% of subjects undergoing an ileocolonic anastomosis with conventional limited mesenteric excision would develop surgical recurrence at 60 months after surgery. To detect a 20% decrease in the surgical recurrence rate after wide mesenteric excision with 80% power and 5% significance, a total of 100 patients must be recruited. The sample size is adjusted to 116 to allow for a 10% loss to follow-up and 5% anastomotic leak.

### Statistical analysis

Data will be presented as median (range), mean, and standard deviation for continuous variables, and percentages for categorical and ordinal data. In order to test whether there are differences between the two groups in the reduction of the primary and secondary outcome, Student’s *t* tests will be performed for continuous variables and chi-square or Fisher’s exact test for categorical variables. Those variables in which statistically significant differences are found will be introduced in the analyses as covariates. Kaplan-Meier analysis with log-rank statistics will be used to assess event-free interval and Cox forward conditional proportional hazards for regression analysis. We plan intention-to-treat analysis of the primary outcome. The full analysis set and per-protocol set will be used for the assessment of efficacy. Interim analyses will be done annually for the first 5 years after index surgery, and the sample size would be adjusted accordingly. An interim analyses will be performed to see if the trial should be continued or stopped, and so it serves as a safeguard. If the outcomes are very poor, more than would be expected, a clear-cut trend towards increased severe morbidity (classed as Clavien-Dindo grade 3 or greater) in relation to either the control or test group, the trial would be stopped and the enrolled participants would have been followed for outcomes and the primary analysis would have focused on the mortality end point. Alternatively, if there was a clear difference between the two groups in terms of benefits for the test cohort, with statistically significant findings emerging on comparison of one group over another that support uncontestable differences between groups, then it might also be stopped and all enrolled participants will be followed for outcomes and the primary analysis will focus on the efficacy end point. Data will be monitored by the treating clinicians and external independent trial monitoring committee, and the principle investigator of each participative site will take overall responsibility. An external independent trial monitoring committee will also analyze interim anonymized outcome data. All analyses considered a *P* value of ≤ 0.05 as statistically significant.

### Cessation of trial

The trial will stop in three circumstances: (1) once 5 years after first enrollment has been reached; (2) statistically significant findings emerge on comparison of one group over another that support uncontestable differences between groups; and (3) a clear-cut trend is demonstrated toward increasingly severe morbidity (classified as Clavien-Dindo grade 3 or greater) in either treatment arm.

## Discussion

This randomized clinical trial should provide evidence on the effectiveness of extensive mesenteric excision on the recurrence of CD. Our hypothesis is that the inclusion of the mesentery during resection of bowel leads to improvement in the natural history of CD. The primary end point is postoperative surgical recurrence, which is a relatively objective outcome. In addition, endoscopic recurrence and clinical recurrence after surgery, which are associated with reoperation for recurrent CD, will be also evaluated. Given that CD includes a thickened mesentery, which can prevent vessel-sealing devices from achieving adequate hemostasis [18], care will be taken to use previously described techniques for safe division of the mesentery [9].

Previous studies of CD have primarily focused on changes within the intestinal wall associated with the disease and on the accompanying inflammatory processes in the mucosa and submucosa. Wide resection of the margins of macroscopically normal bowel to ensure clearance of all macroscopic, and possibly microscopic bowel disease, does not lead to a decrease in postoperative recurrence [19–21]. Mesenteric tissue, which includes fat, lymphatics, vessels, and nerves, is not considered as a “bystander” in CD [3, 22–24]. We recently reported that the increased visceral fat area is an independent risk factor of postoperative disease recurrence in patients with CD [6]. This has been confirmed by a separate study in another ethnic population [25]. This may be partly explained by the findings that increased mesenteric adipose tissue is associated with complex disease status [26], elevated inflammatory markers, bowel-wall thickening [27, 28], and transmural inflammation [29]. In addition to mesenteric fat, lymphatic alterations, such as increased mesenteric lymphatic-vessel density, are also associated with postoperative disease recurrence [7, 30]. Additionally, bacterial translocation to mesenteric adipocytes and to the mesenteric lymph nodes are common in patients with CD [22], which may lead to an increased production of proinflammatory cytokines and result in perpetual inflamed mesenteric and intestinal inflammation. The cross-talk among stromal cells. such as pre-adipocytes, endothelial cells, fibroblasts, and immune cells, including T-cells and macrophages, suggest that the mesentery play a pivotal role in the disease course of CD [3, 4, 31–33]. These may at least in part, explain the results that mesentery-based surgery is associated with decreased postoperative recurrence.

The assessment of surgical factors including hand-sewn or stapled anastomosis, and extensive versus limited intestinal resection have failed to demonstrate any benefit in preventing postoperative recurrence in patients with CD. However, extensive mesenteric resection appears to confer a benefit in smaller studies. Since mesenteric and mucosal abnormalities develop in tandem, we consider the mesentery transition point as the landmark for intestinal division in the mesocolic-resection approach, thereby reducing the otherwise troublesome bleeding typically encountered when dividing the acutely inflamed mesentery [34]. Focusing on the mesentery, in addition to intestine, may offer a new surgical approach for CD [35]. An anticipated challenge of this trial is the concern that a wide mesenteric resection could lead to hematomas and hemorrhage, particularly in an extremely hostile intra-abdominal operating environment with considerable inflammation, adhesions, and abscess formation [18]. However, both our preliminary data and the paper by Coffey et al*.* indicate that the inclusion of the mesentery during ileocolonic resection is safe [5]. Indeed, a safe technique for division of the mesentery is outlined as part of the trial [9], the root of the mesentery will be avoided and the trial participants monitored closely for any increase in complications with this approach.

If the inclusion of the mesentery in ileocolic resection confers a benefit in reducing postoperative recurrence, a mesentery-based approach may become the new standard for surgical resection of CD.

### Trial status

The protocol reported here is version 1.2, dated 30 October 2018. Trial enrollment began on 1 February 2019 and is anticipated to finish on 31 December 2020.

## Supplementary information

**Additional file 1.** Standard Protocol Items: Recommendations for Interventional Trials (SPIRIT) 2013 Checklist: recommended items to address in a clinical trial protocol and related documents; LME EME.

## Data Availability

Data-sharing is not applicable to this article as there are no data generated in the current study.
